# Evaluation of the interaction between insecticide resistance-associated genes and malaria transmission in *Anopheles gambiae* sensu lato in central Côte d’Ivoire

**DOI:** 10.1186/s13071-021-05079-5

**Published:** 2021-11-20

**Authors:** Rosine Z. Wolie, Alphonsine A. Koffi, Ludovic P. Ahoua Alou, Eleanore D. Sternberg, Oulo N’Nan-Alla, Amal Dahounto, Florent H. A. Yapo, Kpahe M. H. Kanh, Soromane Camara, Welbeck A. Oumbouke, Innocent Z. Tia, Simon-Pierre A. Nguetta, Matthew B. Thomas, Raphael NGuessan

**Affiliations:** 1 Unité de Recherche et de Pédagogie de Génétique, Université Félix Houphouët-Boigny, UFR Biosciences, Abidjan, Côte d’Ivoire; 2 Vector Control Product Evaluation Centre, Institut Pierre Richet (VCPEC-IPR), Bouaké, Côte d’Ivoire; 3 Institut Pierre Richet (IPR), Institut National de Santé Publique (INSP), Bouaké, Côte d’Ivoire; 4 Department of Entomology, Center for Infectious Disease Dynamics, The Pennsylvania State University, University Park, PA USA; 5 Department of Disease Control, London School of Hygiene and Tropical Medicine, London, UK; 6 Université Alassane Ouattara, Bouaké, Côte d’Ivoire; 7 Innovative Vector Control Consortium, IVCC, Liverpool, UK; 8 Department of Vector Biology, Liverpool School of Tropical Medicine, Liverpool, L3 5QA UK

**Keywords:** Resistance, Knockdown resistance gene L1014F mutation, Acetylcholinesterase-1 gene G119S mutation, Malaria transmission, *Anopheles gambiae*, *Anopheles coluzzii*, Côte d’Ivoire

## Abstract

**Background:**

There is evidence that the knockdown resistance gene (*Kdr*) L1014F and acetylcholinesterase-1 gene (*Ace-1*^*R*^) G119S mutations involved in pyrethroid and carbamate resistance in *Anopheles gambiae* influence malaria transmission in sub-Saharan Africa. This is likely due to changes in the behaviour, life history and vector competence and capacity of *An. gambiae*. In the present study, performed as part of a two-arm cluster randomized controlled trial evaluating the impact of household screening plus a novel insecticide delivery system (In2Care Eave Tubes), we investigated the distribution of insecticide target site mutations and their association with infection status in wild *An. gambiae* sensu lato (s.l.) populations.

**Methods:**

Mosquitoes were captured in 40 villages around Bouaké by human landing catch from May 2017 to April 2019. Randomly selected samples of *An. gambiae* s.l. that were infected or not infected with *Plasmodium *sp. were identified to species and then genotyped for *Kdr* L1014F and *Ace-1*^*R*^ G119S mutations using quantitative polymerase chain reaction assays. The frequencies of the two alleles were compared between *Anopheles coluzzii* and *Anopheles gambiae* and then between infected and uninfected groups for each species*.*

**Results:**

The presence of *An. gambiae* (49%) and *An. coluzzii* (51%) was confirmed in Bouaké. Individuals of both species infected with *Plasmodium* parasites were found. Over the study period, the average frequency of the *Kdr* L1014F and *Ace-1*^*R*^ G119S mutations did not vary significantly between study arms. However, the frequencies of the *Kdr* L1014F and *Ace-1*^*R*^ G119S resistance alleles were significantly higher in *An. gambiae* than in *An. coluzzii* [odds ratio (95% confidence interval): 59.64 (30.81–131.63) for *Kdr*, and 2.79 (2.17–3.60) for *Ace-1*^*R*^]. For both species, there were no significant differences in *Kdr* L1014F or *Ace-1*^*R*^ G119S genotypic and allelic frequency distributions between infected and uninfected specimens (*P* > 0.05).

**Conclusions:**

Either alone or in combination, *Kdr* L1014F and *Ace-1*^*R*^ G119S showed no significant association with *Plasmodium* infection in wild *An. gambiae* and *An. coluzzii*, demonstrating the similar competence of these species for *Plasmodium* transmission in Bouaké. Additional factors including behavioural and environmental ones that influence vector competence in natural populations, and those other than allele measurements (metabolic resistance factors) that contribute to resistance, should be considered when establishing the existence of a link between insecticide resistance and vector competence.

**Graphical Abstract:**

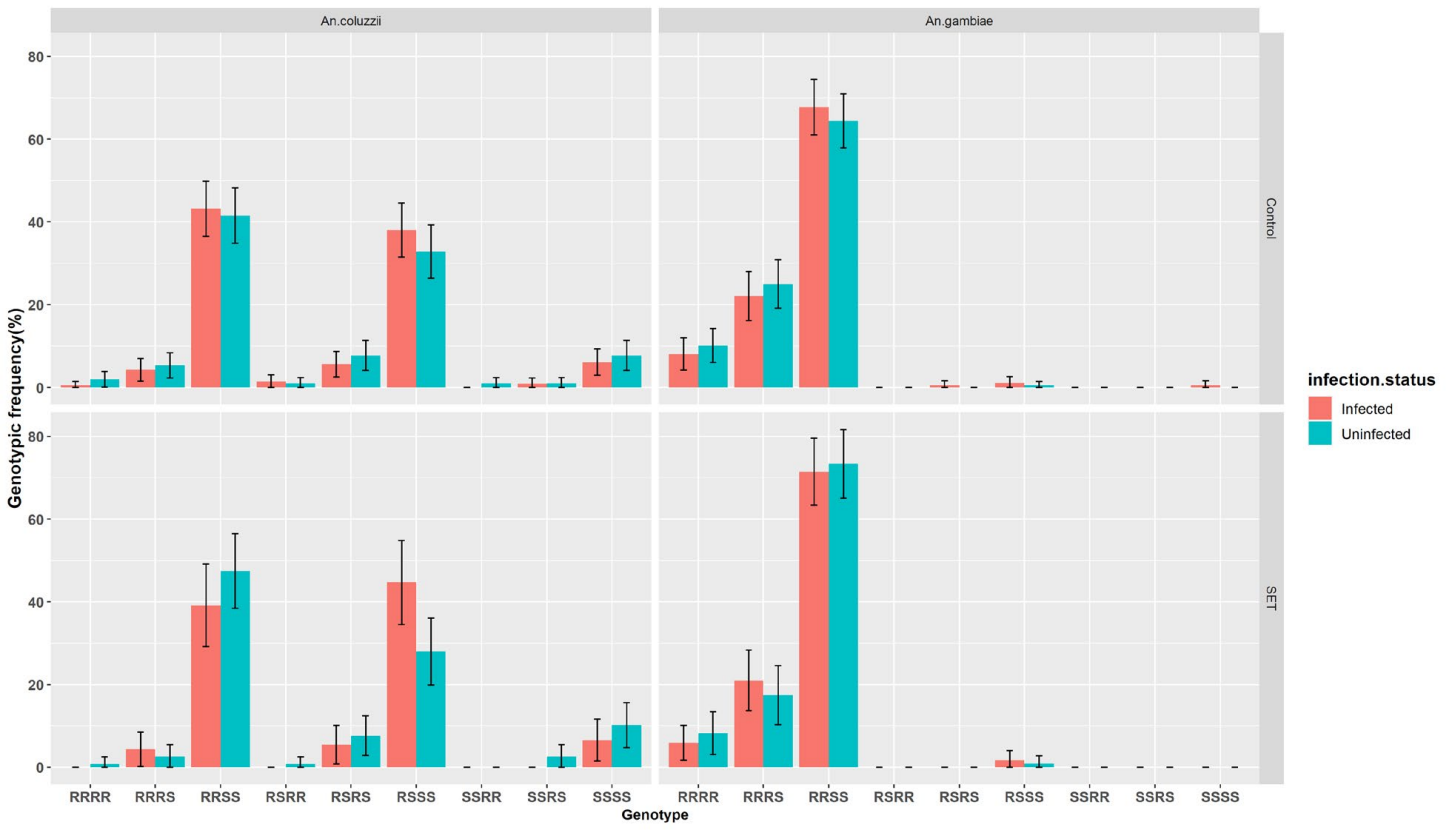

## Background

Mosquitoes of the *Anopheles gambiae* species complex are the main malaria vectors in sub-Saharan Africa [1]. The remarkable vector capacity of these mosquitoes [2] is largely due to their propensity to blood feed on humans and rest indoors [3]. The great ability of these mosquitoes to adapt to human behaviour has led to the development of insecticide-based vector control measures targeting indoor biting and resting. These measures primarily comprise the use of long-lasting insecticidal nets and indoor residual spraying, which are used to limit human-vector contact and reduce mosquito survival [4]. These insecticide-based vector control tools have been highly effective against malaria vectors, as shown by considerable reductions in disease burden [5]. However, the long-term effectiveness of both of these strategies is threatened by the emergence of insecticide resistance in malaria vector populations [6, 7].

There are several mechanisms responsible for insecticide resistance, of which metabolic and target site resistance are the most common [8–10]. Metabolic resistance leads to an increase in the activities of enzymes responsible for an insecticide’s degradation, while modification of the insecticide target site prevents the insecticide molecule from binding to the site. The molecular basis of resistance mediated by target site mutations has been characterized for several mosquito populations [11–13]. For example, the G119S mutation in the acetylcholinesterase-1 gene (*Ace-1*^*R*^) (a single amino acid substitution from glycine to serine at locus 119 at the acetylcholinesterase catalytic site) is responsible for organophosphate and carbamate resistance among malaria vectors in West Africa [14]. Likewise, the L1014F mutation of the knockdown resistance (*Kdr*) gene, also called the *Kdr*-west mutation (an amino acid substitution from leucine to phenylalanine in the voltage gated sodium channel gene, at the 1014 locus, typically causing knock down resistance) is responsible for pyrethroid and dichlorodiphenyltrichloroethane resistance in mosquito populations [12].

Despite the rise of insecticide resistance, its operational significance for vector control is controversial. In many instances, insecticide-based tools seem to continue to protect against malaria [15–18], whereas a community trial of long-lasting insecticidal nets clearly demonstrated that resistance is having an impact on their effectiveness [19]. Resistance is dynamic and therefore cannot be randomized to assess its epidemiological impact. Several studies have evaluated the association between single insecticide resistance gene mutations (of *Kdr* or *Ace-1*^*R*^) and vector competence in *An. gambiae* [20–22]. However, these involved laboratory assays utilizing mosquito colonies or wild strains infected with malaria parasites in the laboratory. The coexistence of both *Kdr* and *Ace-1*^*R*^ in wild populations of *An. gambiae* sensu lato (s.l.) is common in west Africa, including Côte d’Ivoire [23, 24]. To our knowledge, the impact of this association on vector competence has never been studied.

We took advantage of a two-arm cluster randomized controlled trial evaluating the impact of household screening plus a novel insecticide delivery system (In2Care Eave Tubes) to capture mosquitoes in study villages around Bouaké by human landing catches, between May 2017 and April 2019. Mosquitoes were identified to species and then genotyped for *Kdr* L1014F and *Ace-1*^*R*^ G119S mutations using quantitative polymerase chain reaction (qPCR) assays, and the frequencies of the two alleles were compared between *Anopheles coluzzii* and *Anopheles gambiae* and then between infected and uninfected groups for each species.

## Methods

### Study area

The trial was conducted from May 2017 to April 2019 in central Côte d’Ivoire. The methodology used in this study has been well described by Sternberg et al. [25]. Briefly, 40 villages within a 60-km radius in the district of Bouaké were identified for inclusion in the study. All the households in the 40 study villages received insecticide-treated nets, while those of half of the study villages (20 villages) also had household screening (S) and In2Care Eave Tubes (ET) installed (SET).

### Mosquito collection and processing

The mosquito-collection process has been previously described by Sternberg et al*.* [25]. Each month during the trial, mosquitoes were sampled by human landing catches (HLC) both indoors and outdoors at four randomly selected houses in each of the 40 study villages. HLC were undertaken from 6 p.m. to 8 a.m. the following day for two consecutive nights during the first 5 months of the trial and then on one night per month until the end of the trial. The collected mosquitoes were sorted and morphologically identified to species using the key described by Gillies and Meillon [26] and counted. All malaria vectors were stored for further analysis, but for the interaction study, only *An. gambiae* s.l., the main malaria vector in Côte d’Ivoire, was considered.

### DNA extraction

PCR assays were used to assess sporozoite prevalence in a monthly random sub-sample of up to 30 female mosquitoes per village. Mosquitoes were identified to sibling species and* Kdr* L1014F and *Ace-1*^*R*^ G119S mutations detected. Genomic DNA was extracted from the head and thorax of individual females using cetyltrimethylammonium bromide, as described by Yahouedo et al. [27].

### Detection of* Plasmodium* infection

*Plasmodium* spp. (*Plasmodium malariae*, *Plasmodium falciparum*, *Plasmodium ovale* and *Plasmodium vivax*) infections were detected by real-time PCR in accordance with Mangold et al. [28]. The primers were synthesized and supplied by Eurofins Genomics (Ebersberg, Germany) and were as follows: forward PL1473F18 (5′-TAA CGA AGA ACG TCT TAA-3′) and reverse PL1679R18 (5′-GTT CCT CTA AGA AGC TTT-3′). The reactions were prepared in a total reaction volume of 10 μl, which contained 2 μl of 5× HOT FIREPol EvaGreen qPCR Mix Plus (Solis Biodyne, Tartu, Estonia), 0.3 μl of each primer, 6.4 μl of sterile water, and 1 μl of DNA template. The real-time PCR mixtures were pre-incubated at 95 °C for 12 min followed by amplification for 50 cycles of 10 s at 95 °C, 5 s at 50 °C and 20 s at 72 °C, with fluorescence acquisition at the end of each cycle. Characterisation of the PCR product was performed with melting curve analysis of the amplicons (95 °C for 60 s, 60 °C for 60 s, then 60–90 °C for 1 s), with fluorescence acquisition at each temperature transition. *Plasmodium* species were identified by melting curve generated at different temperatures (i.e., for *P. malariae*, 73.5–75.5 °C; for *P. falciparum*, 75.5–77.5 °C; for *P. ovale*, 77.5–79.5 °C; and for *P. vivax*:, 79.5–81.5 °C).

### Species identification

A subsample of 1392 *An. gambiae* s.l. (686 infected with *Plasmodium* sp. and 706 uninfected, which were randomly selected) was analysed for molecular identification of sibling species. The molecular identification was performed using a classic PCR assay in accordance with Favia et al. [29]. The following primers were used: R3 (5’-GCC AAT CCG AGC TGA TAG CGC-3’), R5 (5’-CGA ATT CTA GGG AGC TCC AG-3’), Mopint (5’-GCC CCT TCC TCG ATG GCA T-3’) and B/Sint (5’-ACC AAG ATG GTT CGT TGC-3’). The reaction mixture consisted of 14 μl of sterile water, 0.75 μl of each primer R3 and R5, 1.5 μl of each primer Mopint and B/Sint, and 5 µl of Master Mix. A 23.5-µl volume of the reaction mixture was inserted into each 0.5-ml PCR tube along with 1 µl of each DNA sample. Amplification was performed on a MJ Research PTC-100 Thermal Cycler PCR machine (Marshall Scientific, Watertown, MA) with cycling conditions of 95 °C for 3 min, followed by 30 cycles at 95 °C for 30 s, 72 °C for 45 s and 72 °C for 60 s. Amplified fragments were analysed on 2% agarose gel with 4 μl of SYBR Green. The results were analysed as described in Favia et al. [29] to determine *An. coluzzii* [1300-bp band (R3/R5) plus 727-bp band (Mop-int)] or *An. gambiae* [1300-bp band (R3/R5) plus 475-bp band (B/S-int)].

### Detection of* Kdr* L1014F mutation in* An. gambiae* s.l.

Detection of the *Kdr* L1014F mutation was performed using the TaqMan real-time PCR assay, as described by Bass et al. [30]. The reactions were carried out in a total reaction volume of 10 μl, which contained 2 μl of 5× HOT FIREPol Probe Universal qPCR Mix (Solis Biodyne), 0.125 µl primer/probe mix, 6.875 μl of sterile water, and 1 μl of DNA template.

Primers Kdr-forward (5'-CATTTTTCTTGGCCACTGTAGTGAT-3') and Kdr-reverse (5'-CGATCTTGGTCCATGTTAATTTGCA-3') were standard oligonucleotides with no modification. The probes were labelled with two distinct fluorophores: VIC to detect the susceptible allele, and FAM to detect the resistant allele. Amplifications were performed on a LightCycler 96 Systems real-time qPCR machine (Roche LifeScience, Meylan, France) with cycling conditions of 95 °C for 10 min, followed by 45 cycles at 95 °C for 10 s, 60 °C for 45 s and 72 °C for 1 s. FAM and VIC fluorescence was captured at the end of each cycle and genotypes were called from endpoint fluorescence using LightCycler 96 software (Roche LifeScience) for the analysis of the results.

### Detection of ***Ace-1***^***R***^ G119S mutation in ***An. gambiae*** s.l.

Allelic and genotypic frequencies for insensitive acetylcholinesterase phenotypes characterized by the G119S mutation were determined for *An. gambiae* s.l. by using the TaqMan assay, in accordance with Bass et al. [31]. The reactions were carried out in a total reaction volume of 10 μl, which contained 2 μl of the 5× HOT FIREPol Probe Universal qPCR Mix (Solis Biodyne), 0.125 µl primer/probe mix, 6.875 μl of sterile water, and 1 μl of DNA template. Primers Ace-1-Forward (5’-GGC CGT CAT GCT GTG GAT-3’), and Ace-1-Reverse (5’-GCG GTG CCG GAG TAG A-3’) were standard oligonucleotides with no modification. The probes were labelled with two distinct fluorophores: VIC to detect the susceptible allele and FAM to detect the resistant allele. Amplifications were performed on a LightCycler 96 Systems real-time qPCR machine (Roche LifeScience) with cycling conditions of 95 °C for 10 min, followed by 55 cycles at 92 °C for 15 s, 60 °C for 60 s and 72 °C for 1 s. FAM and VIC fluorescence was captured at the end of each cycle and genotypes were called from endpoint fluorescence using LightCycler 96 software (Roche LifeScience) for the analysis of the results.

### Statistical analysis

To analyse the distribution of *Kdr* L1014F and *Ace-1*^*R*^ G119S genotypic and allelic frequencies, data collected for the same study arm between May 2017 and April 2019 were compared between the species. The association between genotypic and allelic frequencies for these mutations and infection status were determined using Pearson’s chi-square test in R (version 4.0.3). *Kdr* L1014F and *Ace-1*^*R*^ G119S combined genotypic distribution frequencies within infection status for each species were also included. Fisher’s exact test was used when the number of individual samples available for a test was less than 30. The significance threshold was set at 5%. Odds ratio (OR) was computed to assess the strength of difference or association between resistance alleles and infection status. The allelic frequencies were tested to Hardy–Weinberg equilibrium (HWE) conformity using the exact HW test, and were calculated as follows:

$$R\,allelic\, frequency=\frac{RS+2(RR)}{2(RS+RR+SS)}$$

where RR indicates the resistant homozygous genotype, RS the heterozygous genotype, and SS the susceptible homozygous genotype. Nota bene: *Kdr* L1014F and *Ace-1*^*R*^ G119S mutations each comprise three genotypes expressing different allelic variants on the targeted loci. The resistant (R) and susceptible (S) alleles are possible versions of these genes.

### Ethical clearance

Ethical approval was obtained from the ethics committee of the Côte d’Ivoire Ministry of Health (reference 039/MSLS/CNER-dkn), the Pennsylvania State University Human Research Protection Program under the Office for Research Protections (references STUDY00003899 and STUDY00004815), and the ethical review board of the London School of Hygiene and Tropical Medicine (no. 11223). Verbal and written informed consent, using the language spoken locally, was obtained from all the participants (mosquito collectors and head of each household) prior to their enrolment in the study. Mosquito collectors were vaccinated against yellow fever, and the project provided treatment of confirmed malaria cases free of charge for any study participant, in accordance with national policies.

## Results

### Genotypic and allelic frequency distributions of* Kdr* L1014F and* Ace-1*^***R***^ G119S mutations in* An. gambiae* s.l.

**Fig. 1:**
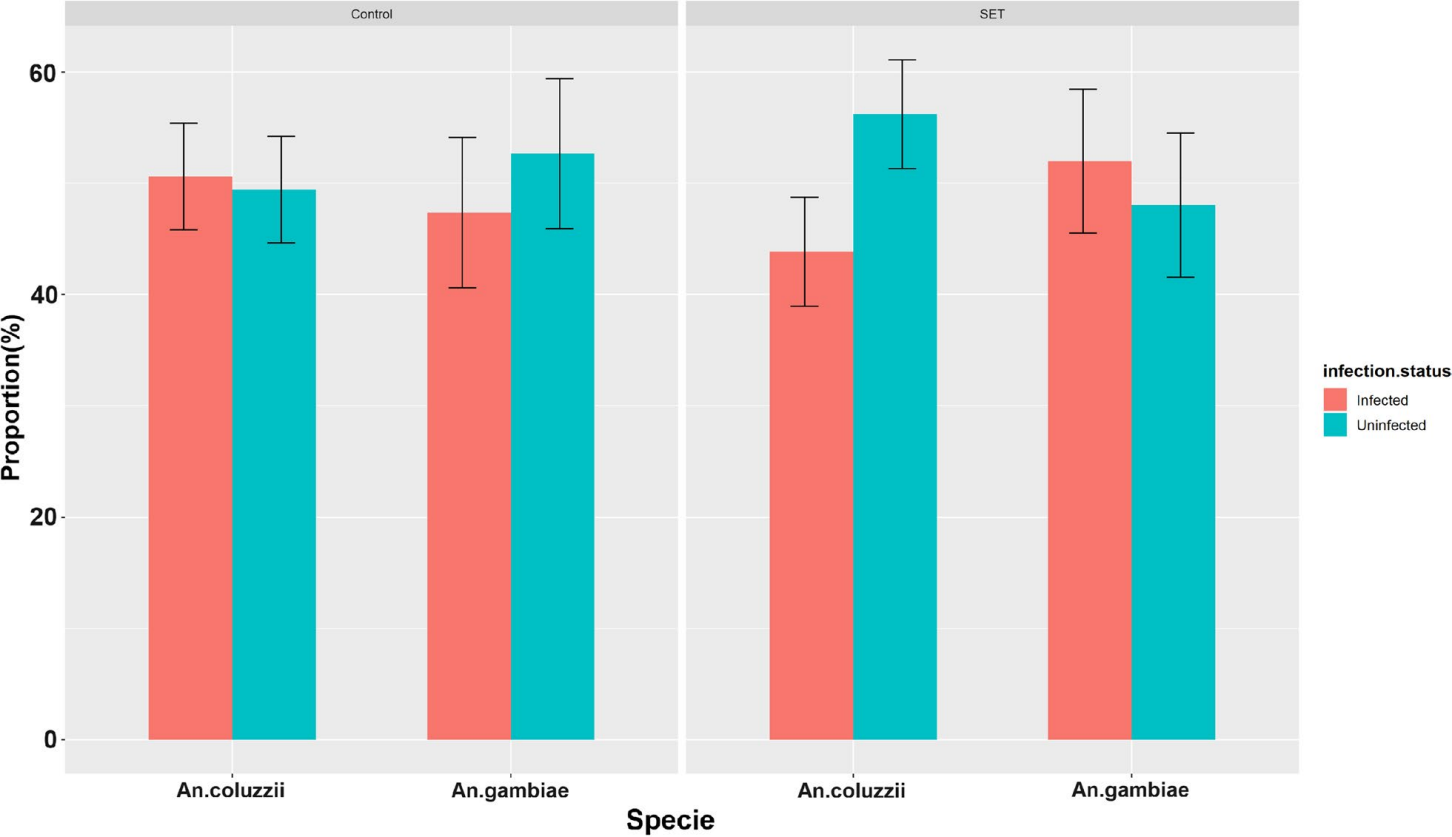
*Anopheles gambiae* sensu lato distribution by infection status.* Error bars* represent 95% confidence intervals (CIs).* SET* Screening plus In2Care Eave Tubes

Out of the 1392 mosquitoes analysed by PCR, 1255 were successfully identified to species (< 10% failure rate). Both *An. gambiae* (*n* = 624; 49.7%) and *An. coluzzii* (*n* = 631; 50.3%) were found. For each species, the proportions of infected vs uninfected individuals were similar (Fig. 1). There were no significant differences in the allelic frequency of *Kdr* or *Ace-1*^*R*^ between the control and Eave Tube areas for each species (*P *˃ 0.05) (Table 1). Genotypic and allelic frequencies of *Kdr* L1014F and *Ace-1*^*R*^ G119S mutations for *An. coluzzii* and *An. gambiae* are shown in Table 2. *Kdr* allelic frequency was significantly greater in *An. gambiae* than in *An. coluzzii* [OR (95% confidence interval; 95% CI): 59.64 (30.81–131.63)] (Table 2). By contrast, the frequency of heterozygous individuals was significantly higher for *An. coluzzii* (42.95%) than for *An. gambiae* (1.12%), indicating deviation from HWE in the *An. gambiae* populations with an excess of resistant homozygous genotypes (Table 2) (*P* < 0.001).

**Table 1:** Allelic frequencies of knockdown resistance gene (*Kdr*) L1014F mutation and acetylcholinesterase-1 gene (*Ace-1*^*R*^) G119S mutation between study arms

		*n*	*Kdr* L1014F				χ^2^ (*P*-value)	*n*	*Ace-1*^*R*^ G119S				χ^2^ (*P*-value)
			SS	RS	RR	R (%)			SS	RS	RR	R (%)	
*Anopheles coluzzii*	Control	421	35	182	204	70.10	0.15 (0.69)	420	356	52	12	9.05	1.79 (0.195)
	SET	210	21	89	100	68.81		210	184	24	2	6.67	
*Anopheles gambiae*	Control	395	1	4	390	99.24	3.87 × 10^–28^ (1)	394	264	94	36	21.07	3.29 (0.069)
	SET	229	0	3	226	99.34		228	168	44	16	16.67	

*n* Number of mosquitoes,* SET* screening plus In2Care Eave Tubes,* SS* susceptible homozygous genotype, *RS* heterozygous genotype,* RR* resistant homozygous genotype,* R* resistant

**Table 2:** Genotypic and allelic frequencies of *Kdr* L1014F and *Ace*-*1*^*R*^ G119S gene mutations in *Anopheles gambiae* and *Anopheles coluzzii*

SNP per species	*n*	Genotypic frequencies^a^ [*x* (%)]			Allelic frequencies [*y* (%)]		OR (95% CI)	HWE χ^2^^b^ (*P*-value)
		RR	RS	SS	R	S		
*Kdr* L1014F								
*An. coluzzii*	631	304 (48.18)	271 (42.95)	56 (8.87)	879 (69.65)	383 (30.35)	1	0.105 (0.744)
*An. gambiae*	624	616 (98.72)	7 (1.12)	1 (0.16)	1232(99.28)	9 (0.72)	59.64 (30.81–131.63)	6.96 (0.008)
*Ace-1*^*R*^G119S								
*An. coluzzii*	630	14 (2.22)	76 (12.06)	540 (85.72)	104 (8.25)	1156 (91.75)	1	23.66 (**< **0.001)
*An. gambiae*	622	52 (8.36)	138 (22.19)	432 (69.45)	250 (20.10)	994 (79.90)	2.79 (2.17–3.60)	51.48 (**< **0.001)

* SNP* Single nucleotide polymorphism,* n* number of mosquitoes, *x* number of genotypes, *y* number of alleles*, OR* odds ratio,* CI* confidence interval,* HWE* Hardy–Weinberg equilibrium, *S* susceptible; for other abbreviations, see Table 1

^a^For the genotypic frequency distribution, values were significantly different (*P*** < **0.001) between *An. coluzzii* and *An. gambiae*

^b^*df* = 2

The allelic frequency of the *Ace-1*^*R*^ G119S mutation was low in both *An. coluzzii* and *An. gambiae*, although it was significantly more prevalent in *An. gambiae* than in *An. coluzzii* [OR (95% CI): 2.79 (2.17–3.60)]. Deviation from HWE for *Ace-1*^*R*^ G119S was observed for both *An. gambiae* and *An. coluzzii* populations.

### Insecticide-resistance genes and infection status

The genotypic and allelic frequencies of *Kdr* L1014F and *Ace-1*^*R*^ G119S gene mutations among infected and uninfected mosquitoes are shown in Table 3. Regardless of the species, there were no significant differences in genotypic or allelic frequencies between infected and uninfected individuals (*P* ˃ 0.05) (Table 3).

**Table 3:** Genotypic and allelic frequencies of *Kdr* L1014F and *Ace-1*^R^ G119S gene mutations between infected and uninfected *Anopheles gambiae* and *Anopheles coluzzii*

Species	Study arm	SNP/status	*n*	Genotypic frequencies [*x* (%)]			Allelic frequencies [*y* (%)]		OR (95% CI)
				RR	RS	SS	R	S	
		*Kdr* L1014F							
*An. coluzzii*	Control	Infected	213	102 (47.89)	96 (45.07)	15 (7.04)	300 (70.42)	126 (29.58)	1
		Uninfected	208	102 (49.04)	86 (41.35)	20 (9.62)	290 (69.71)	126 (30.29)	1.03 (0.76–1.38)
	SET	Infected	92	40 (43.48)	46 (50.00)	6 (6.52)	126 (68.48)	58 (31.52)	1
		Uninfected	118	60 (50.85)	43 (36.44)	15 (12.71)	163 (69.07)	73 (30.93)	0.97 (0.62–1.5)
*An. gambiae*	Control	Infected	187	183 (97.86)	3 (1.60)	1 (0.53)	369 (98.66)	5 (1.35)	1
		Uninfected	208	207 (99.52)	1 (0.47)	0 (0)	415 (99.76)	1 (0.24)	0.17 (0.003–1.6)
	SET	Infected	119	117 (98.32)	2 (1.68)	0 (0)	236 (99.16)	2 (0.84)	1
		Uninfected	110	109 (99.1)	1 (0.9)	0 (0)	219 (99.55)	1 (0.45)	0.53 (0.009–10.4)
		*Ace-1*^*R*^G119S							
*An. coluzzii*	Control	Infected	213	4 (1.88)	23 (10.80)	186 (87.32)	31 (7.28)	395 (92.72)	1
		Uninfected	207	8 (3.86)	29 (14.01)	170 (82.13)	45 (10.87)	369 (89.13)	0.64 (0.38–1.06)
	SET	Infected	92	0 (0)	9 (9.78)	83 (90.22)	9 (4.89)	175 (95.11)	1
		Uninfected	118	2 (1.69)	15 (12.71)	15 (85.60)	19 (8.05)	217 (91.95)	0.58 (0.22–1.40)
*An. gambiae*	Control	Infected	186	15 (8.06)	42 (22.58)	129 (69.35)	72 (19.32)	300 (80.64)	1
		Uninfected	208	21 (10.10)	52 (25.00)	135 (64.90)	94 (22.60)	322 (77.40)	0.82 (0.57–1.17)
	SET	Infected	119	7 (5.88)	25 (21.01)	87 (73.11)	39 (16.39)	199 (83.61)	1
		Uninfected	109	9 (8.26)	19 (17.43)	81 (74.31)	37 (16.97)	181 (83.03)	0.95 (0.56–1.62)

For the genotypic frequency distribution, values between infected and uninfected groups did not differ significantly (*p*** > **0.05). For abbreviations, see Tables 1 and 2

### Frequencies of *Kdr* and* Ace-1*^***R***^ genotypic combinations and infection status

**Fig. 2:**
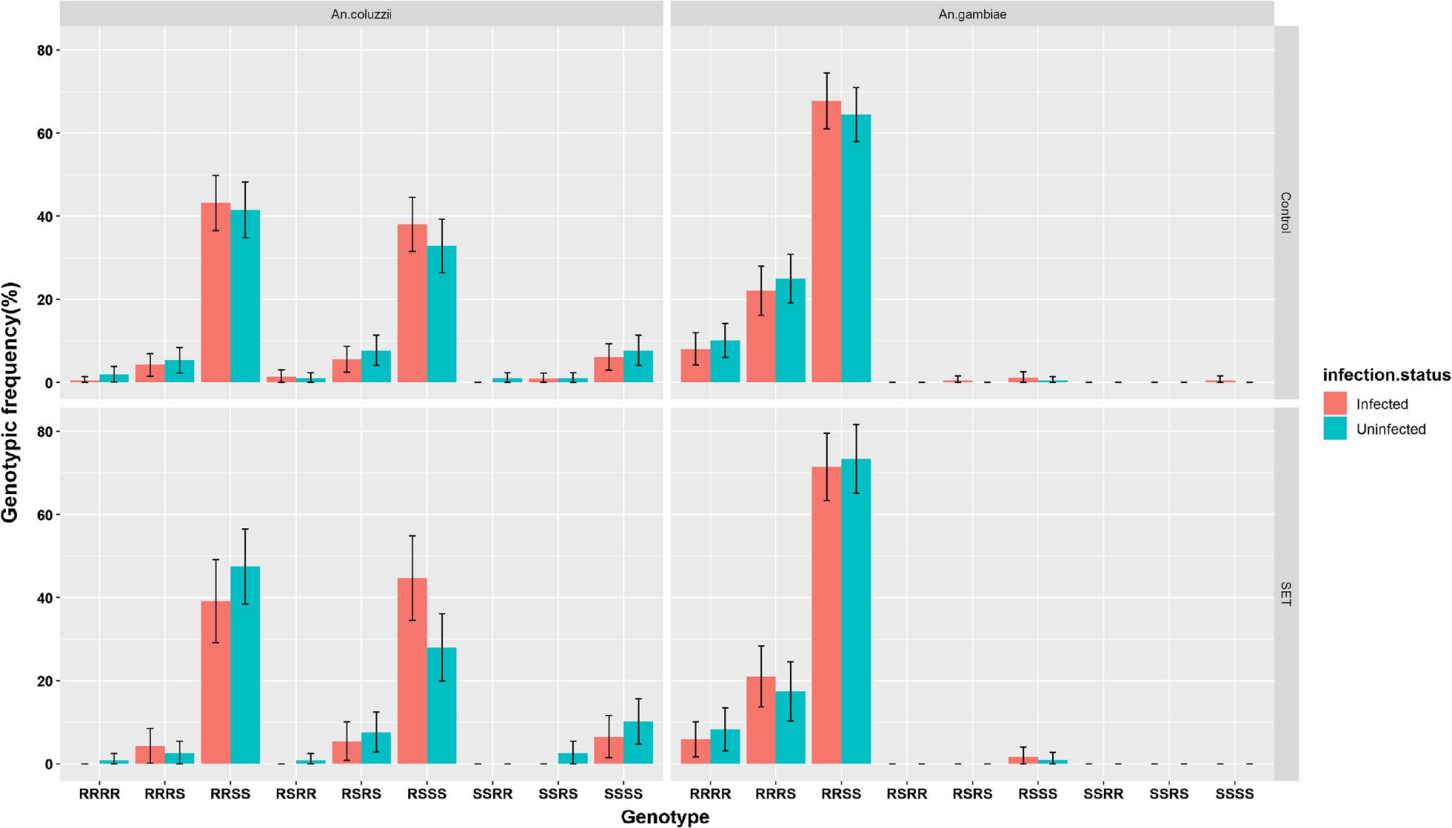
Frequencies of *Kdr* and* Ace-1*^*R*^ genotypic combinations between infected and uninfected groups in each study arm.* Error bars* represent 95% CIs. For all combined genotypes, the first two alleles refer to* Kdr* genotypes and the last two refer to* Ace-1*^*R*^ genotypes. *RR* Resistant homozygous genotype, *RS* heterozygous genotype,* SS* susceptible homozygous genotype; for other abbreviations, see Fig. 1

Nine possible genotypic combinations for the *Kdr* L1014F and *Ace-1*^*R*^ G119S mutations were recorded in this study (Fig. 2). For all genotypic combinations, the first two alleles refer to *Kdr* genotypes whereas the last two alleles refer to *Ace-1*^*R*^ genotypes:* Kdr*-*Ace-1*^*R*^ (RRRR), *Kdr*-*Ace-1*^*R*^ (RRRS), *Kdr*-*Ace-1*^*R*^ (RRSS), *Kdr*-*Ace-1*^*R*^ (RSRR), *Kdr*-*Ace-1*^*R*^ (RSRS), *Kdr*-*Ace-1*^*R*^ (RSSS), *Kdr*-*Ace-1*^*R*^ (SSRR), *Kdr*-*Ace-1*^*R*^ (SSRS), and *Kdr*-*Ace-1*^*R*^ (SSSS). Figure 2 shows that the frequency of individuals bearing *Kdr* RR genotypes, either when present alone or together with *Ace-1*^*R*^ genotypes, was significantly higher in wild *An. gambiae* than in wild *An. coluzzii*; this was observed in both control and SET areas. By contrast, the frequencies of mosquitoes bearing the *Kdr* heterozygous genotype were significantly higher for *An. coluzzii* than for *An. gambiae*, confirming that the former species is better adapted to insecticide pressure than the latter one (Fig. 2). Overall, there were no significant differences between infected and uninfected groups for each of the genotypic combinations for *An. coluzzii* or *An. gambiae*.

## Discussion

This study evaluated the effects of the *Kdr* L1014F and *Ace-1*^*R*^ G119S gene mutations on *Plasmodium* spp. infection status in natural *An. gambiae* s.l. populations. The presence of both *An. coluzzii* and *An. gambiae* in similar proportions in this longitudinal study was consistent with the results of previous studies carried out in the area of Bouaké [24, 32], but it contrasts with the results of another study conducted in adjacent areas within Bouaké that found *An. coluzzii* to be predominant [33]. The observed difference is likely due to the study sampling period covering both the rainy and dry seasons in our study compared to the rainy season only in the other study [33]. We observed no difference in infection rate between *An. gambiae* and *An. coluzzii*. This aligns with the results of previous studies conducted in Burkina Faso and Senegal [21, 34], which reported equivalent susceptibility of these species to *Plasmodium*. The results presented here demonstrate that these sibling species are equally competent vectors of malaria in humans in the central region of Côte d'Ivoire.

With regard to resistance genes, there were no significant differences in the allelic frequency of *Kdr* or *Ace-1*^*R*^ between the control and Eave Tube areas regardless of mosquito species. This is because *Kdr* was already close to fixation (> 80%) in *An. gambiae* s.l. species prior to the intervention employing the Eave Tubes [24], leaving a tiny window for further selection. Also, the insecticide deployed in the Eave Tube trial was a pyrethroid (β-cyfluthrin) [35] which could not induce selection pressure on *Ace-1*^*R*^ since this gene is associated with organophosphate and carbamate resistance [14, 24].

We found significantly higher *Kdr* L1014F and *Ace1*^*R*^ G119S genotypic and allelic frequencies in *An. gambiae* than in *An. coluzzii*, which was in agreement with observations of Koukpo et al. [36] in Benin and Zogo et al. [37] in Côte d’Ivoire. There was a 59 times greater probability of encountering the *Kdr* L1014F resistance allele in *An. gambiae* than in *An. coluzzii*, whereas the frequency of individuals heterozygous for *Kdr* L1014F was higher for *An. coluzzii* (42.95%) than for *An. gambiae* (1.12%). These results clearly highlight a deviation from HWE within both malaria vector species for the *Kdr* L1014F mutation. It is possible that evolutionary factors affect mosquito population structure through the excessive use of insecticides. These factors induce the selection of rare and existing mutations in natural populations of both species which later become variably widespread [38].

Furthermore, *Ace-1*^*R*^ G119S allelic frequency was significantly higher in *An. gambiae* than in *An. coluzzii*, although the amplitude was moderate. The low proportion (< 10%) of homozygous resistant (RR) genotypes observed in *An. gambiae* and *An. coluzzii* populations could indicate a high fitness cost associated with the *Ace-1*^*R*^ G119S gene [39, 40]. Conversely, this potential fitness cost associated with *Ace-1*^*R*^ may be counteracted by the duplication of this gene, which induced various heterozygous genotypes by increasing their proportions [41]. Further studies focusing on *Ace-1*^*R*^ genotype distribution, including duplication in *An. gambiae* s.l., are needed. Our study showed that in areas where *Kdr* L1014F and *Ace-1*^*R*^ G119S coexist in *An. gambiae* s.l., the frequency of individuals bearing the *Kdr* L1014F RR genotype appeared to be significantly higher for *An. gambiae* than for *An. coluzzii*. By contrast, the frequencies of those bearing the *Kdr* L1014F heterozygous genotype were significantly higher for *An. coluzzii* than for *An. gambiae*, confirming the trend seen when this genotype is present in isolation. To our knowledge, this is the first study to evaluate the distribution of *An. gambiae* s.l. individuals bearing both of these mutations. The results presented here call for further studies to better understand the genotypic structure of their combinations.

The vector competence in association with resistance genes was investigated. We found no evidence of an association between *Plasmodium* infection status and *Kdr* L1014F or *Ace-1*^*R*^ G119S gene mutations. These results are similar to those found in a study undertaken in Guinea where these target site mutations (*Kdr* L1014F or *Ace-1*^*R*^ G119S) were not associated with *Plasmodium* infection in wild *An. gambiae* [42], but phenotypic resistance was rather associated with infection. By contrast, a study in Tanzania found a link between *Kdr-*east and *Plasmodium* infection in wild *An. gambiae* [43].

The lack of an association between *Plasmodium* infection status and resistance genes under natural conditions contrasts with the findings of several other studies, which reported that resistance-associated genes affect vector competence for the transmission of *Plasmodium* parasites [20, 21, 44]. There are three possible reasons for these differences. First, these contrasting results could derive from studies that used colonies maintained in the laboratory over years, which can decrease resistance, including a loss of genetic diversity [45, 46]. Second, some genetic susceptibility studies do not take into account additional factors that influence competence in natural vector populations, e.g. mosquito blood-feeding rate, age at infection, longevity, and exposure to an insecticide and to other pathogens that could influence mosquito immune status [47–51]. A natural infection study also implies that the effects of ecology and behaviour on vector competence have been assessed [52, 53]. Third, resistance involves mutations and metabolic components with different functions; therefore studying one in isolation from the other may not be representative of phenotypic resistance. The absence of an association between a combination of genotypes (*Kdr* L1014F-*Ace-1*^*R*^ G119S) and infection status in *An. coluzzii* or *An. gambiae* needs to be considered further in the context of control programmes, given that this is now a common observation in many parts of west Africa [13, 24].

## Conclusions

We found no significant association between the *Kdr* L1014F and *Ace-1*^*R*^ G119S mutations, when present alone or together, and infection status in wild *An. gambiae* and *An. coluzzii*, which demonstrates the similar competence of these species for *Plasmodium* transmission within areas of Bouaké. However, the frequencies of the *Kdr* and *Ace-1*^*R*^ genotypes and alleles were significantly higher in *An. gambiae* than in *An. coluzzii*. Additional factors that influence vector competence in natural vector populations and measurements of factors besides alleles or genotypes that contribute to resistance should be considered when investigating the existence of a link between insecticide resistance and vector competence.

## Data Availability

The data supporting the conclusions of this manuscript are included within the manuscript and are available from the corresponding author on reasonable request.

## Abbreviations

*Ace-1*^*R*^: Acetylcholinesterase-1 gene
CI: Confidence interval
HWE: Hardy–Weinberg equilibrium
*Kdr*: Knockdown resistance gene
OR: Odds ratio
qPCR: Quantitative polymerase chain reaction
SET: Screening plus In2Care Eave Tubes
SNP: Single nucleotide polymorphism
